# Isoflurane Suppresses Proliferation, Migration, and Invasion and Facilitates Apoptosis in Colorectal Cancer Cells Through Targeting miR-216

**DOI:** 10.3389/fmed.2021.658926

**Published:** 2021-08-11

**Authors:** Zhe Cai, Liangyuan Suo, Zeqing Huang

**Affiliations:** Department of Anesthesiology, Cancer Hospital of China Medical University, Liaoning Cancer Hospital & Institute, Shenyang, China

**Keywords:** isoflurane, colorectal cancer, miR-216, proliferation, apoptosis, migration, invasion

## Abstract

**Objective:** Surgery is the first line treatment of colorectal cancer (CRC). Anesthetic isoflurane may improve outcomes of cancer surgery. Herein, we investigated the effects of isoflurane on malignant behaviors of CRC cells and its underlying therapeutic target.

**Methods:** SW620 and HCT116 CRC cells were exposed to a series of concentrations of isoflurane. CCK-8 assay was utilized for determination of the optimal concentration of isoflurane. Under treatment with isoflurane, proliferation, migration, and invasion were separately assessed via clone formation and transwell assays. Apoptotic levels were observed via flow cytometry and expression of Bax, Bcl-2, and Caspase3 proteins was quantified through western blot. MiR-216 expression was detected in isoflurane-induced SW620 and HCT116 cells by RT-qPCR. Following transfection with miR-216 mimic, malignant biological behaviors were examined in isoflurane-treated SW620 and HCT116 cells.

**Results:** 40 μM isoflurane distinctly restrained proliferative, migrated, and invasive capacities and elevated apoptotic levels in SW620 and HCT116 cells. Up-regulation of miR-216 was found in CRC cells. Its expression was suppressed by isoflurane. MiR-216 mimic ameliorated the reduction in proliferation, migration, and invasion and the increase in apoptosis for 40 μM isoflurane-induced SW620 and HCT116 cells.

**Conclusion:** Isoflurane, a promising drug of CRC, may suppress malignant biological behaviors of tumor cells. Furthermore, miR-216 is an underlying target of isoflurane. Thus, isoflurane could be adopted for CRC treatment.

## Introduction

Colorectal cancer (CRC) becomes the third most common malignant tumor as well as the second major cause of cancer-related deaths (1). It was estimated that there were 1,800,977 diagnosed cases and 861,663 death cases globally in 2018 (2). It has been under expectation that death toll will rise in colon and rectal carcinoma by 60 and 71.5% until 2035 (3). The incidence of CRC tends to be younger (4). Though the 5-year survival rate is up to 90% among subjects diagnosed as stage I, this decrease is mildly >10% among those diagnosed as stage IV (5). Distant metastases contribute to unfavorable outcomes of CRC patients. CRC therapies often contain surgery, chemotherapy as well as targeted therapy. However, the therapeutic strategies against CRC remain dissatisfactory. Exploring new drugs and targets is indispensable.

Because CRC patients present a wide range of drug resistance and side effects for various chemotherapy drugs, it is of necessity for screening high-efficient and low-toxic anti-tumor drugs and finding effective therapeutic targets (6). Isoflurane is an inhaled anesthetic widely applied in surgery. Experimental and clinical research has confirmed the varying effects of anesthetics including isoflurane on cancer outcomes (7). Studies have shown that isoflurane is involved in various biological processes like proliferative, apoptotic, invasive, and metastatic capacities for multifarious malignant tumor cells including CRC (8–10). For instance, volatile anesthetic decreases invasive ability of CRC cells via down-regulating MMP-9 (11). Nevertheless, this effect of isoflurane on malignant behaviors of CRC cells and molecular mechanism remains ambiguous.

The progress of CRC is under the coordinated regulation of multiple genes, forming a long-term and complex regulation process (12). Various miRNAs are involved in mediating this disease (13). Identifying individual gene expression differences in CRC is a prerequisite for individualized therapy strategy (14). Recent studies have disclosed various specific miRNAs in tumor tissue specimens and serum from CRC subjects (15–17). Given that miRNAs have disease and tissue specific expression and huge regulatory potential on tumor malignant biological behaviors, miRNAs have aroused widespread concern. Previous studies have reported the roles of miR-216 in several cancers. For instance, miR-216 mediates proliferation, invasion, and cell cycle of gastric cancer cells (18). Furthermore, miR-216 is involved in tumorigenesis of cervical cancer cells (19). Specifically, circulating miR-216 is a predictor of the chemosensitivity for CRC subjects (20). Nevertheless, biological functions including cell proliferation, migration, and invasion of miR-216 in CRC require further exploration. Here, this study found that isoflurane may distinctly suppress proliferative, migrated, and invasive behaviors as well as elevate apoptotic levels of CRC cells partly through targeting miR-216.

## Materials and Methods

### Cell Culture

Human normal colorectal mucosal cells NCM460 and two CRC cell lines SW620 and HCT116 (Cell Resource Center, Shanghai Institute of Biological Sciences, China). Because SW620 and HCT116 cells have been widely applied to study the molecular mechanism and potential drugs of CRC, we chose the two cells in this study. The cells were maintained in RPMI-1640 medium (Gibco, USA) plus 10% FBS in an incubator with 5% CO_2_ at 37°C.

### Quantitative Real-Time Polymerase Chain Reaction (qRT-PCR)

When taking RNA from CRC cells, the culture medium was discarded and rinsed twice. One milliliter of TRIzol reagent (Invitrogen, USA) was added to each well of the six-well plate. Using a pipette to repeatedly aspirate, samples were placed at room temperature to fully dissolve. Then the lysed cell solution was transferred to a centrifuge tube and 0.2 ml chloroform was added to the solution for 10 s. After leaving it at room temperature for 5 min, the cells were centrifuged at 10,000 g at 4°C for 15 min. The upper aqueous phase was transferred to a new RNase-free centrifuge tube. 0.5 ml isopropanol was added and then place it at room temperature for 10 min. Under centrifugation at 10,000 g at 4°C for 5 min, the supernatant was discarded. Cells were treated with 1 ml 75% ethanol made up of DEPC, and centrifuged at 10,000 g at 4°C for 5 min. After discarding the supernatant, 20 μl RNase-free water was added to dissolve the RNA precipitate. Using an ultraviolet spectrophotometer, the absorbance of RNA at OD260 and OD280 was detected. The ratio of OD260/OD280 was utilized for measuring RNA purity. qRT-PCR was used to detect the expression of miR-216. Poly (A) tailing reaction and reverse transcription reaction system were as follows: 5 μl 2 × miRNA reaction buffer mix, 1 μl 0.1% BSA, 1 μl miRNA PrimeScript RT enzyme mix, 2.5 μl RNase-free H_2_O and 0.5 μl total RNA. The reaction system of qRT-PCR was as follows: 20 μl SYBR Premix Ex Taq TMII, 3.2 μl upstream primer, 3.2 μl downstream primer, 4 μl cDNA template. miR-216: 5′-GCCGGCGCCCGAGCTCTGGCTC-3′ (forward), 5′-CATTATTACTTTTGGTACGCG-3′ (reverse), U6: 5′-CTCGCTTCGGCAGCACA-3′ (forward), 5′-AACGCTTCACGAATTTGCGT-3′ (reverse). The qRT-PCR reaction was carried out on the LightCycler 480II qRT-PCR instrument (Roche, Switzerland). The reaction conditions were 95°C for 30 s; at 95°C for 5 s of 40 cycles; at 60°C for 20 s. MiR-216 expression was quantified with the 2^−ΔΔCt^ method.

### Transfection

CRC cells that were in a good growth state and in the logarithmic growth phase were digested with trypsin and inoculated in a 6-well plate. Transfection was presented when the cell density reached 50–60% after 24 h of culture. Fifty nanomolar of miR-216 mimic and negative control (NC; Gene Pharma, China) were transfected into CRC cells. According to the product instruction manual, miRNA mimic and lipofectamine 2000 (Invitrogen, USA) were diluted with Opti-MEM (Invitrogen, USA), which were added to the culture well. After culturing for 4 h, the medium was discarded and the medium containing 10% FBS was replaced to continue the culture. Forty-eight hours after transfection, the cells were collected for qRT-PCR.

### Cell Counting Kit-8 (CCK-8)

CRC cells were seeded into 96-well culture plates (3 × 10^3^ cells per well). They were treated with 0, 5, 10, 20, 40, 80, 160, and 320 μM isoflurane. After culturing for 48 h, each well was replaced with a new medium. Ten microliters of CCK-8 solution (Dojindo, Japan) was added to each well, and continued incubating for 1 h. The microplate reader (Thermo, USA) was utilized to detect the absorbance value of each well at 450 nm wavelength.

### Clone Formation Assay

1 × 10^4^ CRC cells were seeded in 6-well plates. The cells were transfected with miRNA NC and miRNA mimic, respectively. After 48 h, cells were digested with 0.25% trypsin. They were seeded in a 6-well plate (400 cells/well) and cultured for 14 days. The cell culture medium was discarded. The cells were stained with crystal violet at room temperature for 15 min, and slowly washed twice with running water to wash away the dyeing solution. The number of colonies was counted.

### Flow Cytometry

The transfected cells were digested with trypsin digestion solution without EDTA. The cell suspension was centrifuged at 1,000 g for 5 min. After discarding the supernatant, the cells were suspended in PBS and centrifuged again. The supernatant was then discarded. One hundred and ninety-five microliters of Annexin V-FITC (Beyotime, Hangzhou, China) binding solution was added to resuspend the cells, followed by 5 μl Annexin V-FITC and 10 μl PI (Beyotime, Hangzhou, China) staining solution. Flow cytometry was utilized to detect cell apoptosis.

### Western Blot

After 48 h of cell transfection, the culture medium in the 6-well plate was discarded. One hundred and fifty microliters of RIPA lysis buffer was added to each well. The cells were pipetted with a 200 μl pipette. After the cells were fully lysed, the lysate was centrifuged at 14,000 g for 5 min. The protein concentration was determined by the BCA method. The protein sample was mixed with the protein loading buffer in proportion and denatured it in boiling water for 5 min. According to the measured protein concentration, the sample load per well was 20 μg. The protein sample was separated by SDS-PAGE and transferred to PVDF membrane. The membrane was blocked with skim milk at room temperature for 1 h. After rinsing the membrane with 1 × TBST for 5 min × 3 times, the membrane was incubated with primary antibody against Bax (1:1,000; ab270742; Abcam, USA), Bcl-2 (1:1,000; ab196495; Abcam, USA), Caspase3 (1:1,000; ab90437; Abcam, USA) and GAPDH (1:1,000; ab181602; Abcam, USA) overnight at 4°C. The membrane was placed on a 1 × TBST horizontal shaker and rinsed for 10 min × 3 times. HRP-labeled secondary antibody (ab7090; Abcam, USA) was diluted to 1:2,000 with 5% blocking solution and incubated it on a shaker at room temperature for 1 h. After rinsing again, the membrane was incubated with ECL reagent (Beyotime, Hangzhou, China). Image J analyzed the optical density value of the target band.

### Transwell

Transwell assays were utilized for detecting migration and invasion. For migration assay, transwell chamber (Corning Costar, USA) was coated without Matrigel. For invasion assay, transwell chamber was coated with Matrigel (BD, USA). 3 × 10^5^ CRC cells were seed onto the upper chamber. The lower chamber was incubated with 600 μl RPMI-1640 medium plus 20% FBS at 37°C. Using a cotton swab, cells that did not migrate or invade the upper chamber were removed. The cells in the lower chamber were fixed with methanol for 10 min and stained with crystal violet (Beyotime, Hangzhou, China) solution for 5 min.

### Sample Collection

Totally, 30 CRC tissues and matched adjacent normal tissues were harvested from the Cancer Hospital of China Medical University (China). All of them did not experience chemotherapy/radiotherapy before surgery. Specimens were confirmed by two pathologists as CRC. This research was in line with the Declaration of Helsinki. This study acquired the approval of the ethics committee of the Cancer Hospital of China Medical University (2020021). Each participant signed a written informed consent. MiR-216 expression was detected in CRC and control tissues by qRT-PCR.

### Statistical Analysis

Statistical analyses were presented through SPSS 21.0 software (SPSS Inc., Chicago, IL, USA). Data were expressed as the mean ± standard deviation. Comparisons between groups were analyzed by student's *t*-test or one-way ANOVA followed by Tukey's *post-hoc* test. *P* < 0.05 was indicative of statistical significance.

## Results

### Isoflurane Restrains CRC Cell Proliferation

**Figure 1:**
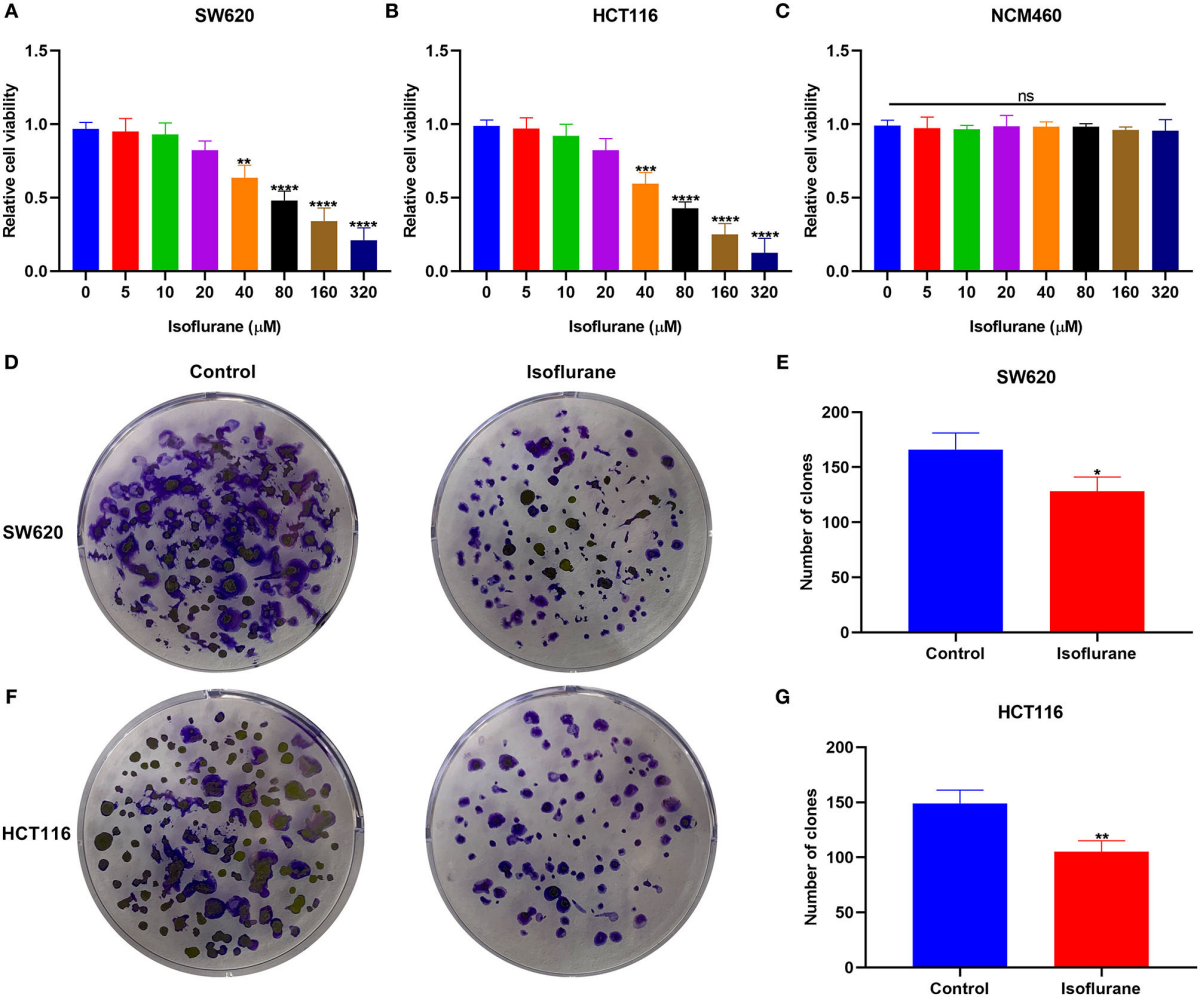
The inhibitory effects of isoflurane on CRC cell proliferation. The cell viability of **(A)** SW620, **(B)** HCT116, and **(C)** NCM460 cells treated by 0, 5, 10, 20, 40, 80, 160, and 320 μM isoflurane. The number of clones of **(D,E)** SW620 and **(F,G)** HCT116 cells under treatment with 40 μM isoflurane. Ns, no significance; ^*^p < 0.05; ^**^p < 0.01; ***p < 0.001; ^****^p < 0.0001.

To determine the optimal concentration of isoflurane on CRC cells, SW620 and HCT116 CRC cells were treated with 0, 5, 10, 20, 40, 80, 160, and 320 μM isoflurane. CCK-8 results revealed that cell viability was inversely proportional to isoflurane concentrations in SW620 and HCT116 cells (Figures 1A,B). Compared to controls, when the concentration of isoflurane was 40 μM, cell viability was distinctly reduced in SW620 (^*^p < 0.01) and HCT116 cells (^***^p < 0.001). Thus, 40 μM isoflurane was determined as the optimal concentration for next assays. More importantly, a series of concentrations of isoflurane did not affect the viability of normal colorectal mucosal cells NCM460 (Figure 1C). When SW620 cells were treated with 40 μM isoflurane, the number of clones showed a distinct shrinkage than controls (^*^p < 0.05; Figures 1D,E). This reduction was confirmed in another CRC cell line HCT116. As shown in Figures 1F,G, 40 μM isoflurane distinctly shrunk the number of HCT116 cell clones (^**^p < 0.01).

### Isoflurane Induces CRC Cell Apoptosis

**Figure 2:**
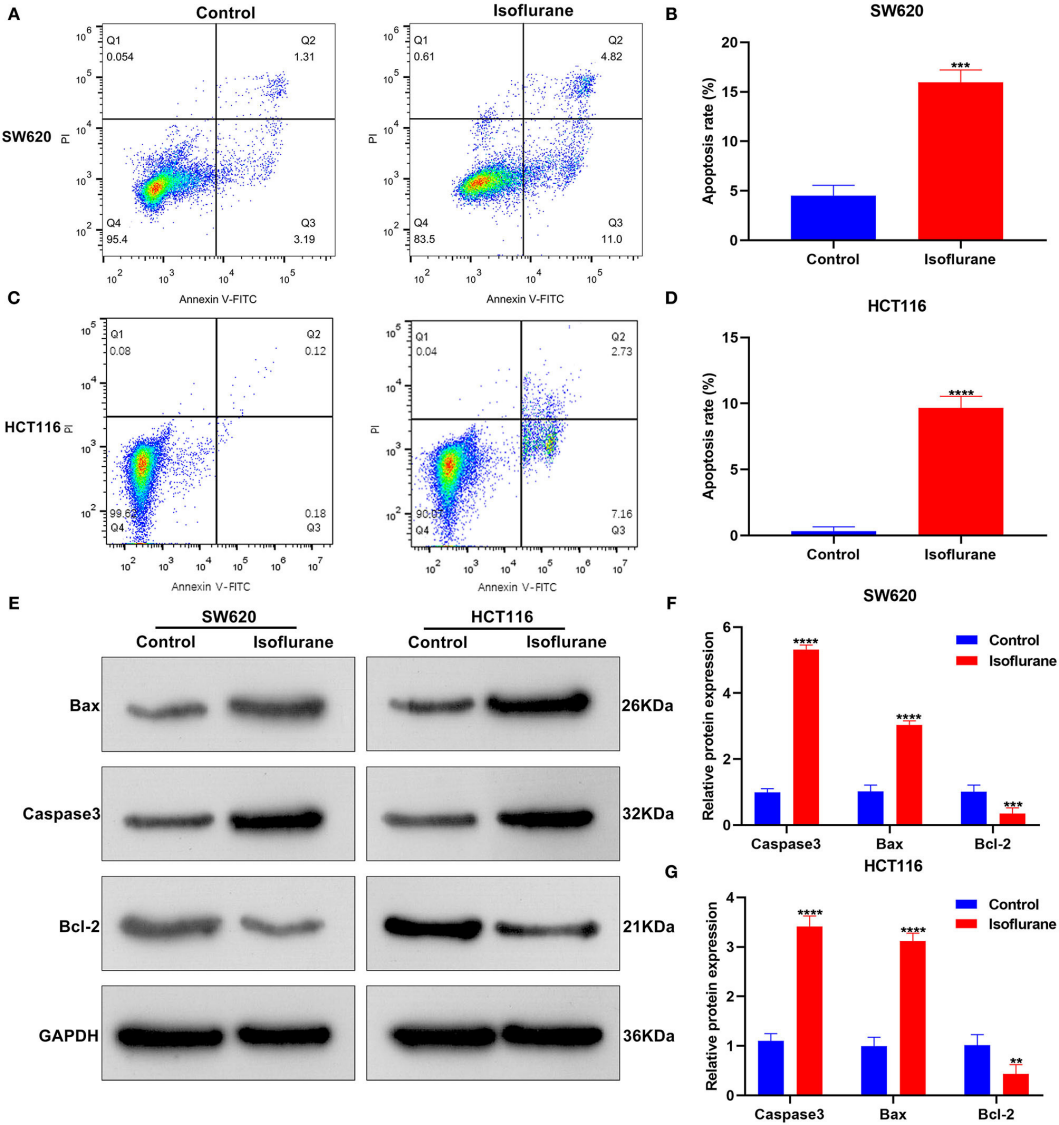
The promotional effects of isoflurane on CRC cell apoptosis. Apoptosis rates of **(A,B)** SW620 and **(C,D)** HCT116 cells under treatment with 40 μM isoflurane. **(E–G)** Expression of Caspase3, Bax and Bcl-2 proteins in SW620 and HCT116 cells following exposure to 40 μM isoflurane. ^**^p < 0.01; ^***^p < 0.001; ^****^p < 0.0001.

We assessed the influence of isoflurane on CRC cell apoptosis. When SW620 cells were exposed to 40 μM isoflurane, flow cytometry data signified that apoptotic rate was significantly increased than controls (^***^p < 0.001; Figures 2A,B). The similar findings were detected in HCT116 cells. In Figures 2C,D, 40 μM isoflurane displayed a promotional effect on HCT116 cell apoptosis (^****^p < 0.0001). Apoptosis-related markers Caspase3, Bax, and Bcl-2 were tested in 40 μM isoflurane-induced SW620 and HCT116 cells. Data were indicative of the increase in Caspase3 (both ^****^p < 0.0001) and Bax (both ^****^p < 0.0001) expression as well as the decrease in Bcl-2 (^***^p < 0.001 and ^**^p < 0.01) expression in SW620 and HCT116 cells under exposure to 40 μM isoflurane (Figures 2E–G).

### Isoflurane Exerts a Suppressive Role on Migrated and Invasive Capacities of CRC Cells

**Figure 3:**
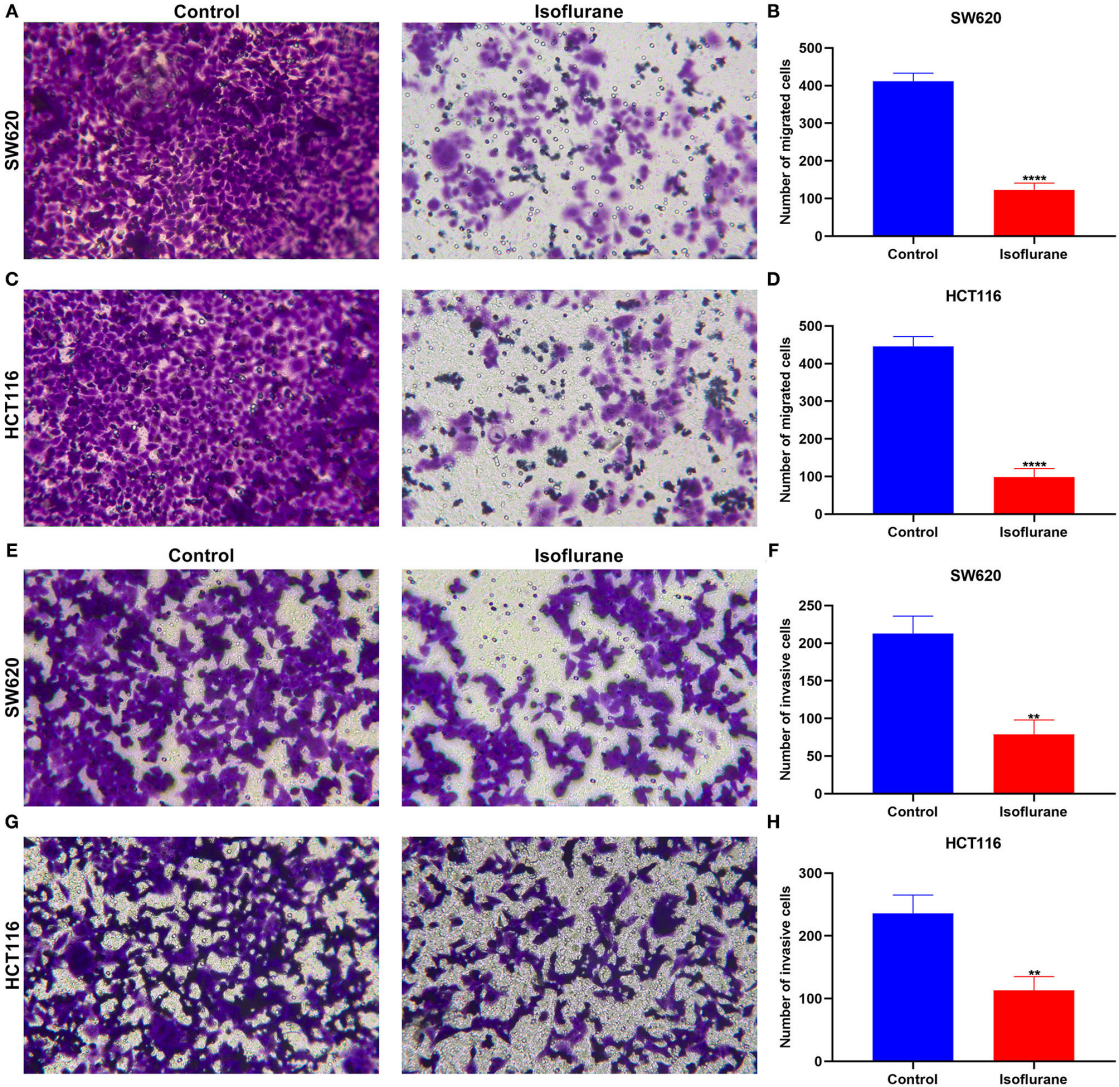
The suppressed role of isoflurane on migrated and invasive capacities of CRC cells. The number of migrated **(A,B)** SW620 and **(C,D)** HCT116 cells following 40 μM isoflurane treatment. The number of invasive **(E,F)** SW620 and **(G,H)** HCT116 cells exposed to 40 μM isoflurane. ^**^p < 0.01; ^****^p < 0.0001.

Migrated and invasive capacities of CRC cells were evaluated under exposure to 40 μM isoflurane. In comparison to controls, the number of migrated SW620 cells was distinctly lessened when treated with 40 μM isoflurane (^****^p < 0.0001; Figures 3A,B). Similarly, 40 μM isoflurane exhibited a suppressive role on HCT116 cell migration (^****^p < 0.0001; Figures 3C,D). Meanwhile, the number of invasive SW620 cells was markedly shrunk under exposure to 40 μM isoflurane (^**^p < 0.01; Figures 3E,F). The suppressive function of isoflurane on HCT116 cell invasion was found in Figures 3G,H (^**^p < 0.01).

### Isoflurane Decreases miR-216 Expression in CRC Cells

**Figure 4:**
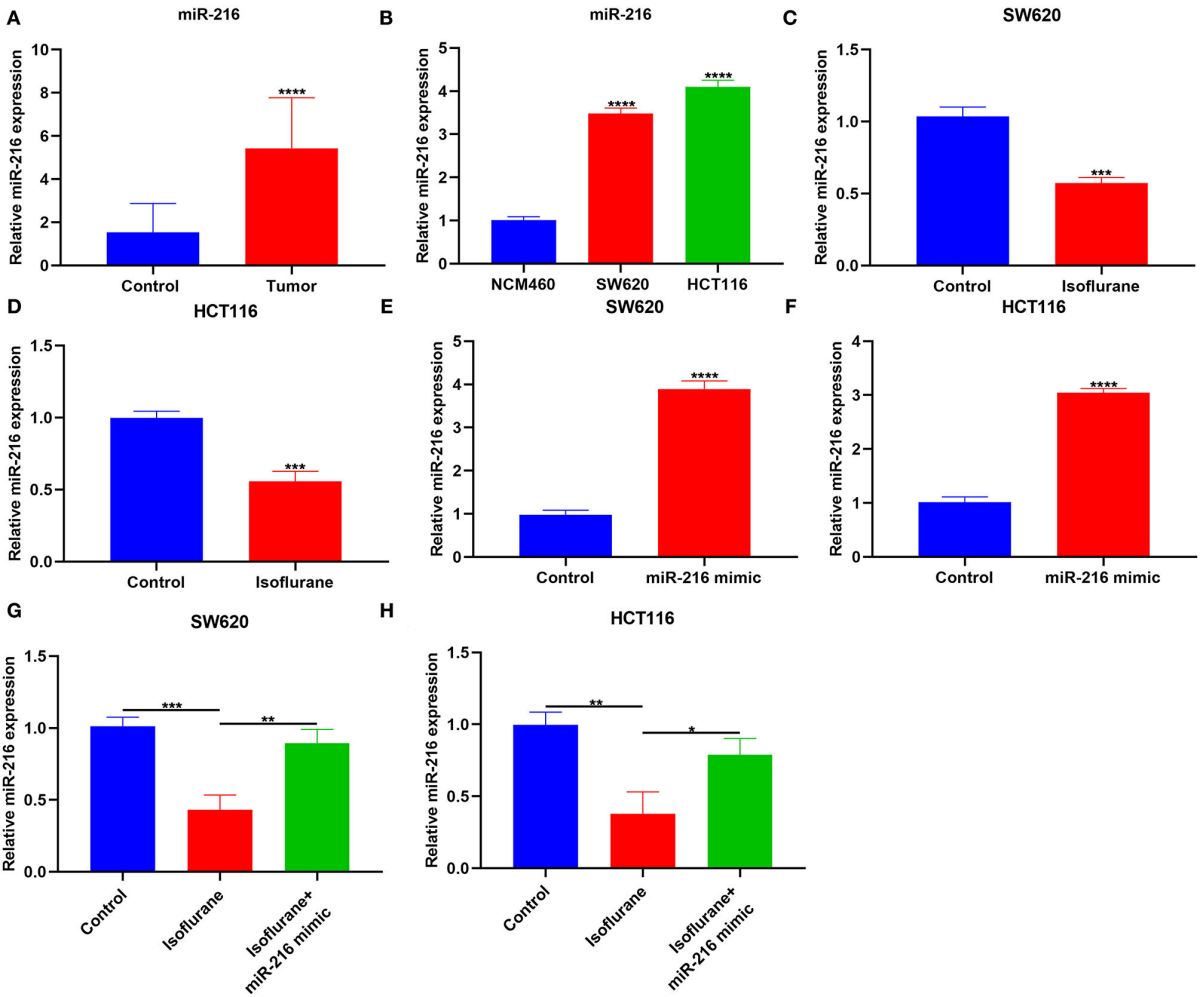
The inhibitory function of isoflurane on miR-216 expression in CRC cells. **(A)** RT-qPCR for miR-216 expression in CRC and control tissue specimens. **(B)** miR-216 expression in NCM460, SW620, and HCT116 cells. **(C,D)** MiR-216 expression in **(B)** SW620 and **(C)** HCT116 cells exposed to 40 μM isoflurane. MiR-216 expression in **(E)** SW620 and **(F)** HCT116 cells transfected by miR-216 mimic. MiR-216 expression in **(G)** SW620 and **(H)** HCT116 cells following treatment with 40 μM isoflurane and/or miR-216 mimic. ^*^p < 0.05; ^**^p < 0.01; ^***^p < 0.001; ^****^p < 0.0001.

This study collected 30 paired CRC and control tissues. Our data showed that miR-216 displayed higher expression in CRC compared to control specimens (^****^p < 0.0001; Figure 4A). Furthermore, miR-216 displayed an increased expression in SW620 and HCT116 cells compared to NCM460 cells (both ^****^p < 0.0001; Figure 4B). Under exposure to 40 μM isoflurane, miR-216 expression had reduced levels in SW620 and HCT116 cells than controls (both ^***^p < 0.001; Figures 4C,D). To further confirm whether miR-216 was a target of isoflurane, miR-216 expression was markedly overexpressed by its mimic in SW620 and HCT116 cells (both ^****^p < 0.0001; Figures 4E,F). Our data were indicative that the decrease in miR-216 expression induced by isoflurane was distinctly ameliorated by co-transfection of miR-216 mimic in SW620 (^**^p < 0.01) and HCT116 cells (^*^p < 0.05; Figures 4G,H). These data were indicative of miR-216 as a target of isoflurane in CRC cells.

### Isoflurane Restrains Proliferation and Facilitates Apoptosis in CRC Cells Partly Through miR-216

**Figure 5:**
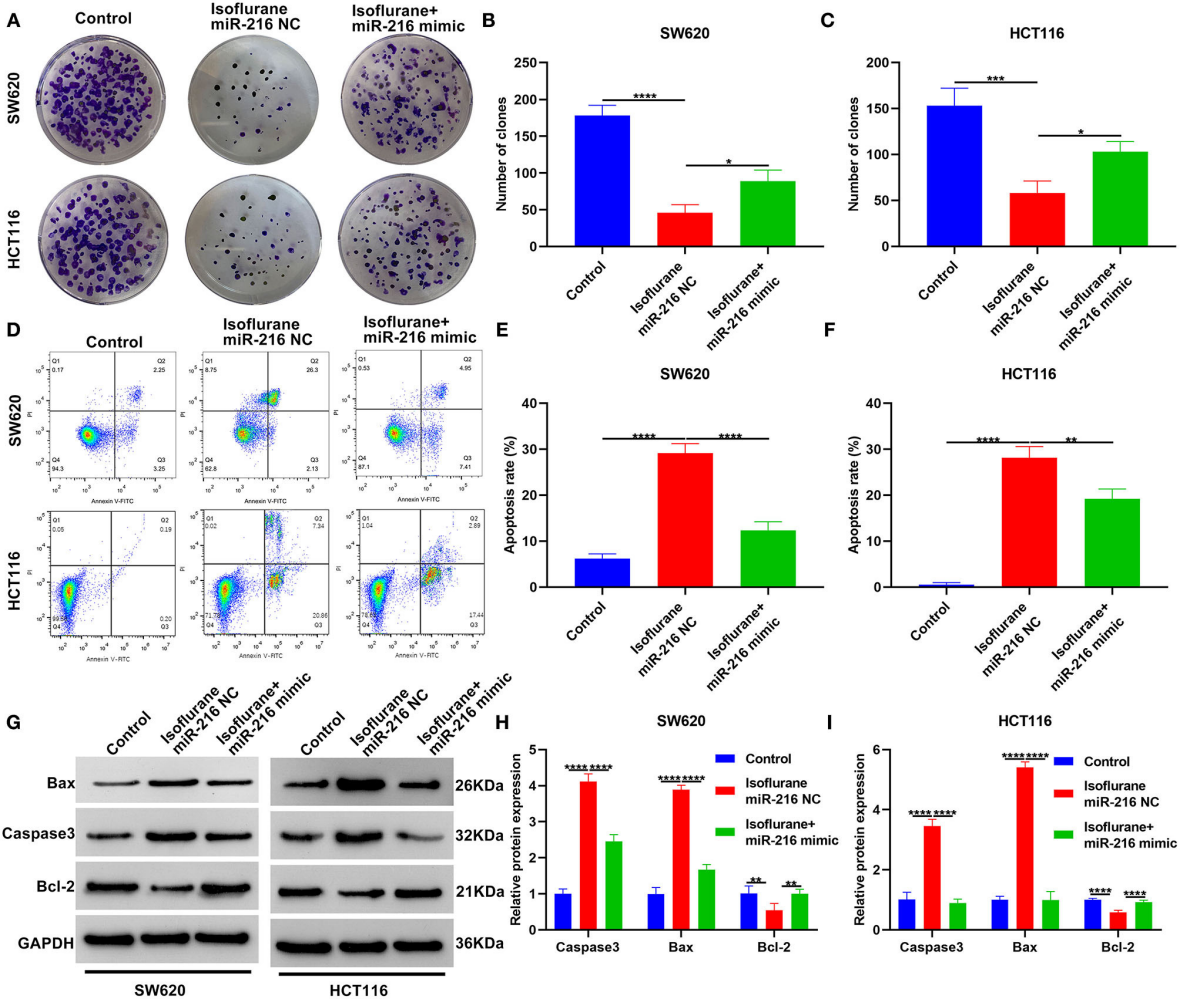
Isoflurane restrains proliferation and facilitates apoptosis in CRC cells partly through miR-216. **(A–C)** The number of clones of SW620 and HCT116 cells treated with isoflurane and/or miR-216 mimic. **(D–F)** Apoptotic rate of SW620 and HCT116 cells under treatment with isoflurane and/or miR-216 mimic. **(G–I)** Expression of Caspase3, Bax and Caspase3 and Bax proteins in SW620 and HCT116 cells following treatment with isoflurane and/or miR-216 mimic. ^*^p < 0.05; ^**^p < 0.01; ^***^p < 0.001; ^****^p < 0.0001.

This study investigated whether isoflurane exerted inhibitory effects on malignant behaviors of CRC cells via suppression of miR-216. We found that miR-216 mimic markedly ameliorated the inhibitory function of isoflurane on SW620 and HCT116 cell proliferation (both ^*^p < 0.05; Figures 5A–C). Apoptotic levels were assessed via flow cytometry. Data demonstrated that the increase in apoptotic levels of 40 μM isoflurane-induced SW620 (^****^p < 0.0001) and HCT116 cells (^**^p < 0.01) was markedly weakened by miR-216 mimic (Figures 5D–F). In Figures 5G–I, the increase in Caspase3 and Bax proteins and the decrease in Bcl-2 protein was markedly ameliorated by miR-216 mimic (all ^****^p < 0.0001). Above data suggested that isoflurane restrained proliferation and facilitated apoptosis in CRC cells partly through miR-216.

### Isoflurane Weakens Migration and Invasion of CRC Cells Partly Through miR-216

**Figure 6:**
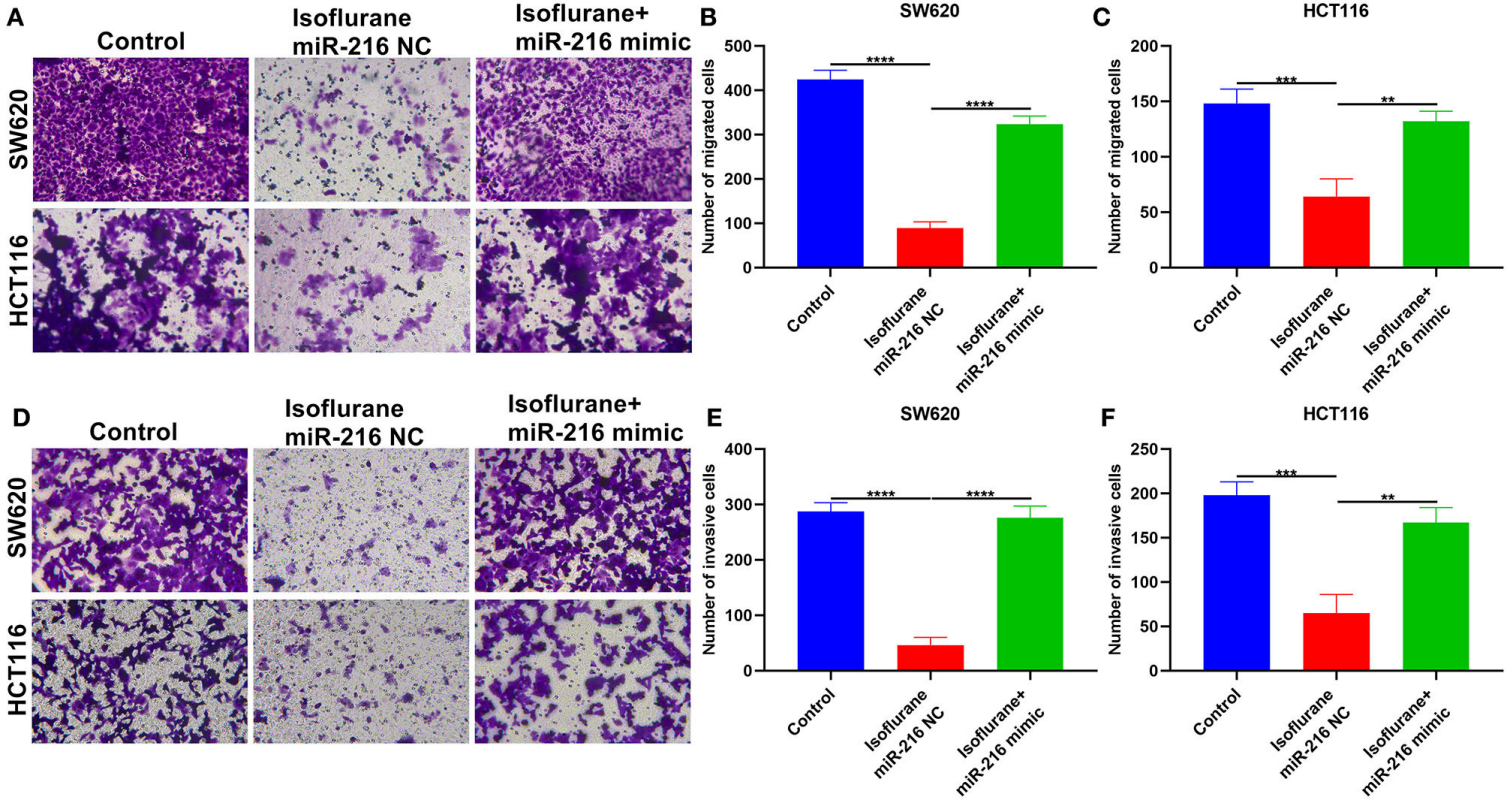
Isoflurane weakens migration and invasion of CRC cells partly through miR-216. **(A–C)** The number of migrated SW620 and HCT116 cells under exposure to isoflurane and/or miR-216 mimic. **(D–F)** The number of invasive SW620 and HCT116 cells following treatment with isoflurane and/or miR-216 mimic. ^**^p < 0.01; ^***^p < 0.001; ^****^p < 0.0001.

**Figure 7:**
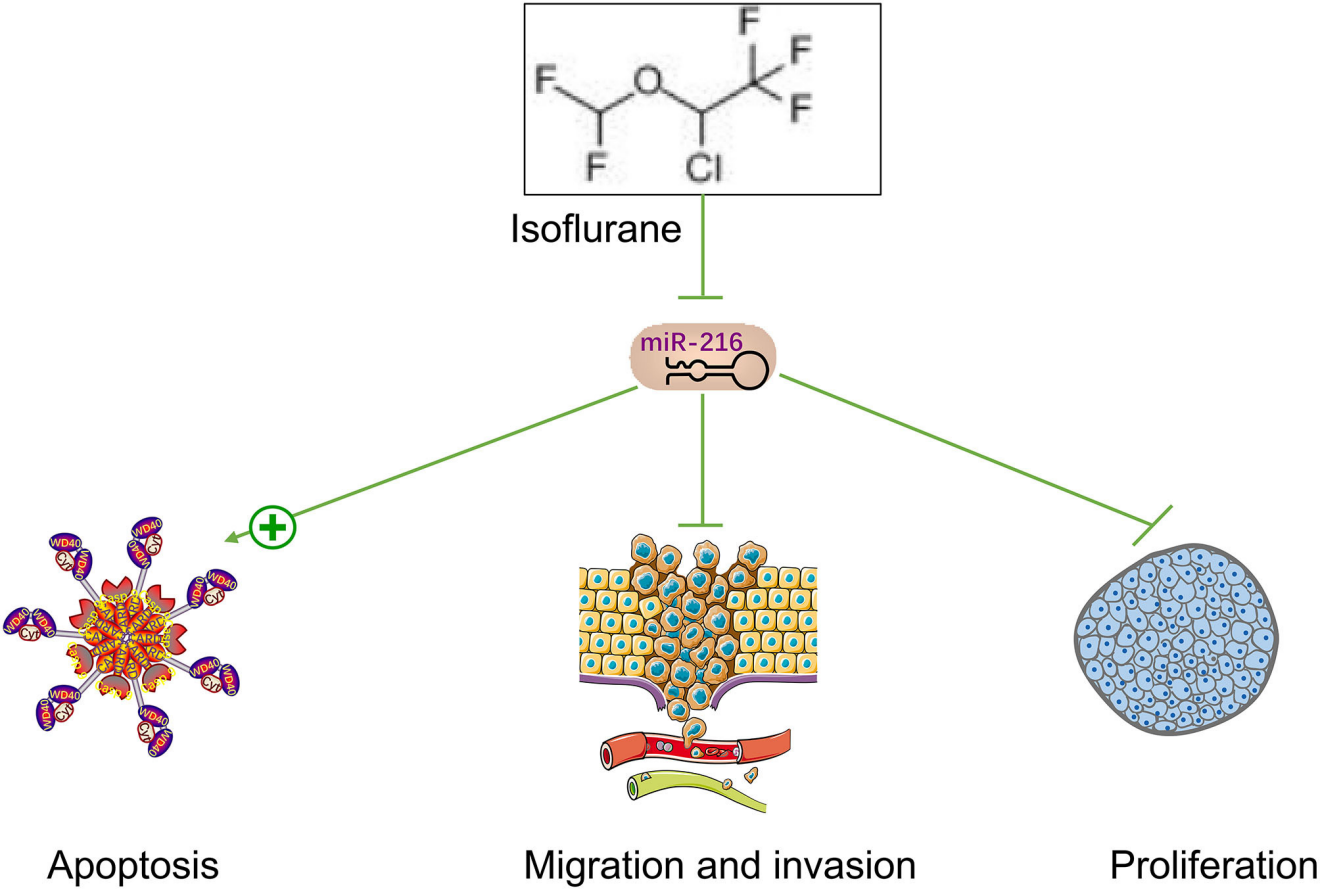
The mechanism diagram of this study.

This study further investigated whether isoflurane exposure could affect migration and invasion of CRC cells via miR-216. Data showed that the decrease in the number of migrated SW620 (^****^p < 0.0001) and HCT116 (^**^p < 0.01) cells exhibited an improvement by co-treatment with miR-216 mimic (Figures 6A–C). Furthermore, miR-216 mimic markedly improved the decrease in the number of invasive SW620 (^****^p < 0.0001) and HCT116 (^**^p < 0.01) cells (Figures 6D–F). Collectively, isoflurane weakened migrated and invasive abilities of CRC cells partly through suppression of miR-216. Figure 7 depicted the schematic diagram of this study.

## Discussion

CRC is a common malignant tumor of the digestive tract, and its occurrence is related to heredity, environment, and lifestyle (21–23). At present, most conventional treatment methods are surgery, radiotherapy, chemotherapy, etc. (24–26). However, postoperative recurrence and metastasis, insensitivity to radiotherapy, and resistance to chemotherapy often occur, and the clinical efficacy is not ideal. Therefore, finding safe and effective treatment methods and new drugs has become a research focus in recent years. This study proposed that isoflurane was an underlying drug agent. Forty micromolar of isoflurane restrained proliferative, migrated, and invasive behaviors as well as elevated apoptotic levels in SW620 and HCT116 CRC cells. MiR-216, as a target of isoflurane, was lowly expressed in CRC cells. The up-regulation lessened the therapeutic effects of isoflurane on CRC. Hence, isoflurane exerted the inhibitory functions on malignant behaviors of CRC through miR-216.

The cell proliferation and apoptosis disorders are the main causes of tumors (27). The main mechanism of action of anti-tumor drugs is to inhibit tumor cell proliferation and induce apoptosis. This study showed that isoflurane displayed the inhibitory effects on CRC cell proliferation in a concentration-dependent manner but did not affect normal colorectal mucosal cells. Invasion and metastases are the most basic biological characteristics of malignancies. CRC has a strong ability of invasion as well as migration. Controlling distant metastases in the liver, lungs, and kidneys is the difficulty and key point in treating CRC. The current treatment methods are still difficult for patients with metastatic CRC. Thus, it is of significance concerning developing effective targeted intervention methods against CRC cell invasion and migration. Here, isoflurane restrained migrated as well as invasive abilities of CRC cells. Hence, isoflurane may be a promising drug against CRC. Recently, Lu et al. found that isoflurane did facilitate invasion as well as metastases of bladder carcinoma cells via HIF-1α/β-catenin/Notch1 axis (9). Zhang et al. reported the enhancement of isoflurane on proliferative ability of squamous cervical carcinoma cells via mTOR-dependent pathway (28). As shown in research by Guo et al., isoflurane displayed the stimulative role on glucose metabolism of ovarian carcinoma cells by up-regulating miR-21 (29).

MiRNAs have been proven as key roles on the biology of CRC. Dysregulated miRNAs are involved in CRC progress by accommodating the expressions of specific target genes. Due to their high stability, miRNAs are also considered valuable biomarkers. Our data were indicative of the up-regulation of miR-216 in CRC cells. miR-216 expression is varying in different tumor specimens. Xiao et al. found that miR-216 motivated proliferative as well as invasive behaviors in bladder carcinoma cells through PIK3R2-regulated PI3K/Akt pathway (30). Chen et al. reported the regulatory functions of miR-216b on cellular proliferation, invasion as well as cell cycle through cyclin T2 in gastric carcinoma cells (18). The study from Zhang et al. confirmed miR-216b expression as an accurate marker of acute myeloid leukemia recurrence (31). Li et al. confirmed the down-regulation of miR-216b in glioma and its overexpression restrained tumor cellular growth as well as migration through activating AEG-1 (32). MiRNAs display closely correlations to CRC tumor growth as well as metastases (33–35). Herein, miR-216 was a target of isoflurane in CRC. Up-regulated miR-216 ameliorated the decrease in proliferation, migration, and invasion and increase in apoptotic levels in isoflurane-induced CRC cells. These data were indicative of the suppressive roles of isoflurane on CRC cellular malignant behaviors partly through miR-216. However, several limitations of this study should be pointed out. First, the possible mechanism that miR-216 is an underlying target of isoflurane requires further exploration. Second, clinical implication of miR-216 should be validated in a large CRC cohort. Third, the inhibitory effect of isoflurane on CRC will be observed *in vivo*.

## Conclusion

Taken together, isoflurane, as a promising drug agent, distinctly restrained proliferative, migrated, and invasive behaviors as well as elevated apoptotic levels in CRC cells. MiR-216 up-regulation was discovered in CRC cells. MiR-216 was an effective target of isoflurane. MiR-216 overexpression may markedly lessen the therapeutic efficacy of isoflurane on CRC cellular malignant behaviors. These data were indicative of the therapeutic potential of isoflurane on CRC. Isoflurane exerted the inhibitory effects on CRC partly through miR-216. More assays require to confirm the therapeutic functions of isoflurane.

## Data Availability Statement

The original contributions presented in the study are included in the article/supplementary material, further inquiries can be directed to the corresponding author/s.

## Ethics Statement

The studies involving human participants were reviewed and approved by This study acquired the approval of the ethics committee of the Cancer Hospital of China Medical University (2020021). The patients/participants provided their written informed consent to participate in this study.

## Author Contributions

ZH conceived and designed the study. ZC conducted most of the experiments, data analysis, and wrote the manuscript. LS participated in collecting data and helped to draft the manuscript. All authors reviewed and approved the manuscript.

## Conflict of Interest

The authors declare that the research was conducted in the absence of any commercial or financial relationships that could be construed as a potential conflict of interest.

## Publisher's Note

All claims expressed in this article are solely those of the authors and do not necessarily represent those of their affiliated organizations, or those of the publisher, the editors and the reviewers. Any product that may be evaluated in this article, or claim that may be made by its manufacturer, is not guaranteed or endorsed by the publisher.

## Abbreviations

CRC: colorectal cancer
qRT-PCR: quantitative real-time polymerase chain reaction
NC: negative control
CCK-8: cell counting kit-8
ns: no significance.
